# Essential Amino Acids in the Gluten-Free Diet and Serum in Relation to Depression in Patients with Celiac Disease

**DOI:** 10.1371/journal.pone.0122619

**Published:** 2015-04-17

**Authors:** Nathalie J. M. van Hees, Erik J. Giltay, Susanne M. A. J. Tielemans, Johanna M. Geleijnse, Thomas Puvill, Nadine Janssen, Willem van der Does

**Affiliations:** 1 Institute of Psychology, Leiden University, Leiden, The Netherlands; 2 Department of Psychiatry, Leiden University Medical Center, Leiden, The Netherlands; 3 Division of Human Nutrition, Wageningen University, Wageningen, The Netherlands; 4 Leyden Academy on Vitality and Ageing, Leiden, The Netherlands; Rosalind Franklin University, UNITED STATES

## Abstract

**Introduction:**

Celiac disease (CD) is associated with an increased risk of major depressive disorder, possibly due to deficiencies in micronutrients in the gluten-free diet. We aimed to investigate whether essential amino acids (i.e., the precursors of serotonin, dopamine and other neurotransmitters) are depleted in the diet and serum of CD patients with major depressive disorder.

**Methods:**

In a cross-sectional study we assessed dietary intake of amino acids and serum levels of amino acids, in 77 CD patients on a gluten-free diet and in 33 healthy controls. Major depressive disorder was assessed with structured interviews (using the Mini International Neuropsychiatric Interview Plus). Dietary intake was assessed using a 203-item food frequency questionnaire.

**Results:**

Participants had a mean age of 55 years and 74% were women. The intake of vegetable protein was significantly lower in CD patients than in healthy controls (mean difference of 7.8 g/d; 95% CI: 4.7–10.8), as were serum concentrations of tyrosine, phenylalanine and tryptophan (all *p* < 0.005). However, within the CD patient group, the presence of major depressive disorder (n = 42) was not associated with intake or serum levels of essential amino acids.

**Conclusions:**

Patients with CD on a long-term gluten-free diet, with good adherence, consume significantly less vegetable protein than controls, and their serum levels of several essential amino acids were also lower. Despite its potential adverse effect, intake and serum levels of essential amino acids were not related to major depression.

## Introduction

Celiac disease (CD) is an immune-mediated intolerance for gluten in genetically predisposed individuals. CD has a prevalence rate in Europe and the USA of about 1:100 [1], and is associated with an increased prevalence of psychopathology, particularly the major depressive disorder [2]. When a patient with CD ingests gluten peptides the T-lymphocytes of the small intestine initiate an inappropriate immune response, resulting in chronic inflammation of the jejunal mucosa, intestinal malabsorption and atrophy of the small intestinal villi. The clinical presentation varies widely, as CD is now considered a multisystem disorder affecting multiple organs, such as the skin, thyroid, heart, nervous system, pancreas, spleen, liver [3], as well as the brain. The only treatment for CD is a lifelong strict gluten-free diet, which leads to restoration of the atrophied intestinal villi. It may, however, take years to achieve complete recovery [4]. The effects of the gluten-free diet on mood and psychiatric symptoms are largely unknown and deserve more research attention, considering that dietary factors may influence psychopathology.

Common psychiatric symptoms seen in untreated CD are anxiety, apathy, irritability, and depressed mood [5–7]. Psychiatric disorders, in particular major depression and panic disorder are also found. One cross-sectional, case-control study in 36 CD patients and 144 controls using the diagnostic interview method to assess life-time psychopathology found an increased prevalence of major depressive disorder (42%), dysthymic disorder (8%), adjustment disorders (31%), and panic disorder (14%) [6]. After they have started treatment with a gluten-free diet, depressive and anxiety symptoms improve but remain prevalent in CD patients [8,9].

In a previous study we have investigated the fatty acid content of the gluten-free diet and related the fatty acid levels in intake and serum to current, remitted and partially remitted depression in CD patients. We found that the fatty acid intake of CD patients on a longstanding successful gluten-free diet was similar to healthy controls and unrelated to having any major depressive disorder [10]. In the present report we investigated the hypothesis that the gluten-free diet is a risk factor for depression in treated CD patients, through the mechanism of amino acid deficiencies, in line with biological theories of depression.

**Fig 1:**
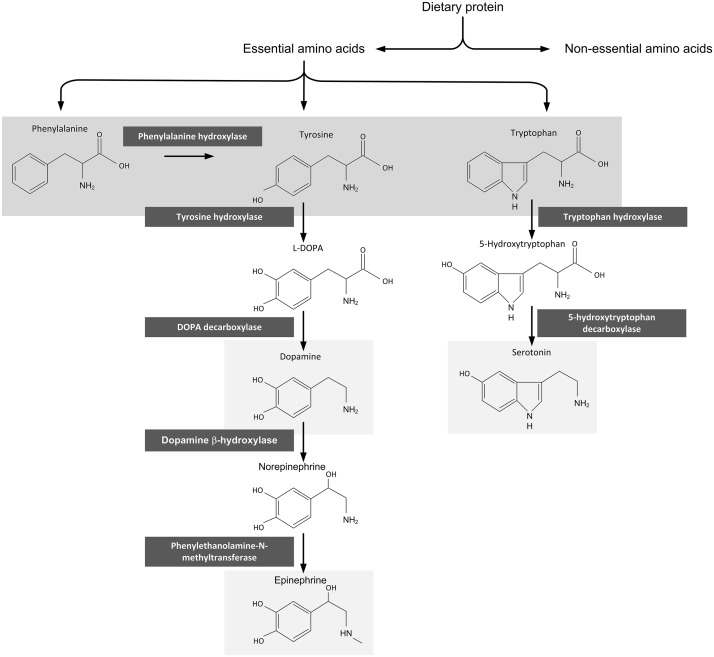
Amino acid metabolism.

Several studies have revealed abnormalities in monoamine metabolism in patients with active CD [7,11,12]. Monoamine neurotransmitters and neuromodulators (i.e. serotonin, dopamine, norepinephrine, and epinephrine) play a role in the regulation of mood and cognitive functioning. Low monoamine function is associated with psychopathology such as anxiety and mood disorders [13]. Two studies investigating the effect of monoamine availability on psychiatric symptoms and disorders in active CD found an inverse correlation [14,15]. Several authors have hypothesized that malabsorption in active CD is linked to brain function and depression via reduced uptake of amino acids such as tryptophan and reduced production of monoamines such as serotonin [2,7,11,14,16]. One study investigated the effect of the introduction of the gluten-free diet in five adolescent CD patients with depression. They found lower pre-diet serum amino acid concentrations and institution of the gluten-free diet produced an improvement in psychiatric symptoms at three month follow-up, with a non-significant increase in tryptophan concentrations and a significant increase in the competing amino acids [17]. Another study, in 15 untreated and 12 treated children with CD and 12 controls, found significantly lower plasma tryptophan and tyrosine concentrations (62% and 21% respectively) compared to controls in treated CD patients at one year follow-up [7]. A study in seven adults with CD showed that gluten-free diet increased cerebrospinal fluid concentrations of major monoamine metabolites. After maintaining a gluten-free diet for a period ranging from 7 to 18 months the cerebrospinal fluid concentrations of the major serotonin metabolite 5-HIAA had a significant increase of 31% [11]. Studies following patients for a longer time have however not been performed and the question remains if concentrations of monoamine metabolites fully return to normal levels in treated CD. An informative overview of amino acid and monoamine metabolism can be found in Fig 1.

The diet has been shown to be low in important nutrients [18–20]. Its restrictive nature may lead to a deficient intake of amino acids and to nutritional deficiency in general. Whether nutritional deficiencies caused by the daily diet contribute to the risk of depression is unknown. However, the daily food intake of CD patients, rich in corn and low in dairy products, resembles the circumstances that are created in experimental acute tryptophan depletion studies. These studies involve the dietary restriction of tryptophan, in combination with the consumption of a large quantity of large neutral amino acids, and this has been shown to have an acute mood lowering effect, in individuals vulnerable to depression, and cognitive effects in most people [21]. The gluten-free diet does contain sources of amino acids, however gluten-free bread is often made of cornflower which is, compared to wheat flour, low in the amino acids lysine and tryptophan and high in other large amino acids such as leucine and valine [22]. L-tryptophan (Trp) is an essential amino acid, which means that it needs to be acquired through the diet. It functions as a precursor of serotonin (and also melatonin and niacin [vitamin B3]). An adult requires about 285 mg per day to maintain nitrogen balance [23]. The amount of Trp per 100 g wheat flour (as in bread) is139 mg, thus by deleting wheat flour from the diet a substantial source of Trp is removed. The other large neutral amino acids (LNAA), i.e. tyrosine (Tyr), phenylalanine (Phen) and the branched-chain amino acids, valine, isoleucine and leucine, compete with Trp at the blood-brain barrier for the same transport mechanism into the brain [24,25]. Because of this competition, a gluten-free diet that is poor in Trp and rich in LNAA could therefore restrict Trp access to the brain, which might consequently affect serotonin concentrations in the brain [26]. Acute Trp depletion has been shown to have a mood lowering effect and to cause relapse in patients with remitted major depressive disorder [21]. We propose that low serotonin concentrations depletion—caused by the gluten-free diet—might play a role in the maintenance and worsening of depression after the start of the dietary treatment. Furthermore, depression may affect dietary choices that may exacerbate the problem.

In the present study we aim to investigate this hypothesis by assessing the intake and plasma levels of the essential amino acid Trp, Phen and Tyr, and their ratio to LNAA in patients with CD with current or past major depressive disorder. We expected that low intake of Trp, Phen and Tyr and lower ratios to LNAA are related to an increased risk of major depressive disorder.

## Methods

### Participants

**Fig 2:**
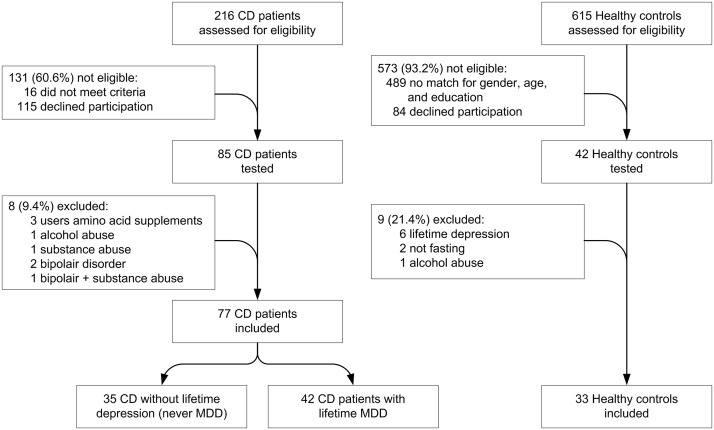
Flow chart of participants in the study.

CD patients were recruited from participants in a previous Dutch survey study [9] who gave permission to be contacted (Fig 2). They were selected on the criterion of maintaining a strict gluten-free diet for 2 years or more. In order to obtain equal group sizes, participants who had expressed the presence of depressive symptoms, were oversampled to form the major depressive disorder group of CD patients. Never-depressed controls without CD were recruited from a general population sample and matched for age, gender and level of education. These participants were recruited through general practitioners, and had been willing to participate in a previous study as a control group to assess normal values for psychiatric scales [27]. A total of 127 participants entered the study (85 CD patients and 42 controls). A more detailed explanation of the recruitment procedure, the study procedures and the measurement instruments has been described elsewhere [10]. Confirmation of CD diagnosis was obtained from medical records for all but 8 (11.2%) participants. The exclusion criteria for all participants in the current study were: age younger than 18, having an inflammatory bowel disease other than CD, or having conditions which would make the testing session unreliable or impossible. CD participants were also excluded if they had low adherence to the gluten-free diet or were on the diet for less than 2 years. Healthy controls where excluded if they had any mood disorder diagnosis, had coeliac disease or were on a gluten-free diet. Seventeen participants were excluded on one or more of the following criteria, resulting in a final sample size of 110 (Fig 1): bipolar disorder (n = 3), current alcohol abuse (n = 2), current drug abuse (n = 1), not fasting on morning of testing (n = 2), healthy control with lifetime diagnosis on Mini International Neuropsychiatric Interview of any mood disorder (n = 6), and the use of Trp dietary supplements (n = 3).

### Procedure and ethics

In accordance with the declaration of Helsinki, this study was approved by the Medical Ethics Committee of Leiden University Medical Centre (ref. nr. P09.131) and all participants provided written informed consent before the start of data collection. Participants had the capacity to consent, as assessed during screening, and there was no surrogate consent procedure. Participants younger than 18 years or with a diminished capacity to give informed consent or participate, were not included in the study. Participants were sent the package with study information, instructions, questionnaires, and an informed consent form two weeks before the day of testing. The testing day started with signing of the informed consent form and a data check on the questionnaires. After that the physical examination and blood collection were performed. Participants had been fasting up to the blood collection and were provided with a light breakfast afterwards. The session concluded with a structured psychiatric diagnostic interview.

### Instruments

#### Psychiatric diagnoses

The complete Mini International Neuropsychiatric Interview Plus 5.0.0-R (MINI-Plus) [28], Dutch version [29], was administered. The MINI-Plus is a structured clinical diagnostic interview of current and lifetime Axis-I disorders according to the criteria of the Diagnostic and Statistical Manual of Mental Disorders—Fourth Edition (DSM-IV) [30]. Interviews were conducted by a trained team. Participants with a mood disorder were categorized in the ‘partially remitted MDD’ group if they recently had an episode of major depressive disorder (MDD) but now had subclinical symptoms. Participants with dysthymia or double depression were placed in the major depressive disorder groups (n = 4 in the ‘current MDD’, and n = 1 in the ‘remitted MDD’ group). Participants from the remitted, partially remitted and current major depressive disorder groups combined form the ‘lifetime MDD’ group.

#### Food Frequency Questionnaire

A validated semi-quantitative food frequency questionnaire that was previously used in epidemiological studies in The Netherlands was used [31–33]. The questionnaire covers the 1-month intake of 203 food items and beverages. For the present study, additional questions on the use of gluten-free food products were added to the food frequency questionnaire and participants were asked to provide the packaging and labels of the gluten-free products that they had used in the past month. We also asked for recipes of homemade gluten-free bread including the weight in grams of an average slice. Nutrient intake was calculated using the Dutch Food Composition Table (‘Nederlands Voedingsstoffenbestand’; NEVO, 2006) [34], which was extended for gluten-free products by a dietician based on the information provided by the participants and inquiry with manufacturers.

#### Other variables

Body mass index (kg/m^2^) was computed. The average amount of physical activity was assessed using the Physical Activity Scale for the Elderly [35], to estimate ‘metabolic equivalents of task’ (MET) minutes. Smoking behavior, alcohol consumption and duration of gluten-free diet was assessed using self-report questionnaires. The current gluten-free diet adherence level in our CD patients was assessed with a self-report question [9] as well as with the Coeliac Disease Adherence Test [36].

#### Blood sampling and other measures

Fasting venous blood samples were obtained on ice, centrifuged and stored at—80°C within 3 hours after collection. Six serum amino acids (valine, isoleucine, leucine, tyrosine, phenylalanine, tryptophan) and serum high-sensitivity C-reactive protein (hsCRP) were assessed. HsCRP concentrations (mg/L) were measured using nephelometry. Valine, isoleucine and leucine were considered to be the branched-chained amino acids (BCAA). Tryptophan, valine, isoleucine and leucine were considered to be the LNAA. Fasting serum amino acid composition was determined as a concentration (μmol/L) on a Biochrom 30 automated amino acid analyser (Biochrom, UK) as previously described [37]. The lower limits of detection were 3.5 μmol/L for valine, isoleucine and leucine; 2.5 μmol/L for tyrosine and phenylalanine, and 6.5 μmol/L for tryptophan. The intra-assay variabilities were 2.1% for valine, 2.3% for leucine, and 1.9% for phenylalanine, while the inter-assay variabilities were 1.8% for valine, 3.1% for leucine and 3.9% for phenylalanine (in n = 10).

### Statistical Analysis

Differences in group means were analyzed using analysis of variance (ANOVA) for continuous variables and chi-squared (χ^2^) tests for categorical variables. Analysis of covariance (ANCOVA) was used to adjusted for the possible confounders age, gender, education, BMI, alcohol intake and smoking in analyses involving dietary intake and to adjust for age, gender, education, BMI, alcohol intake, smoking and total energy intake in analyses involving plasma concentrations. To assess the potential mediation by dietary intake, we additionally adjusted for daily intake of Trp and LNAA when analyzing plasma levels of amino acids in a second ANCOVA. Because of the unequal group sizes the p-value for the Welch’s F statistic is reported for variables in ANOVA where the assumption of homogeneity of variance is violated. Post hoc tests in ANOVA were perfomed using a Games-Howel procedure. Post hoc tests in ANCOVA were performed using a Sidak confidence interval correction for multiple comparisons. Sensitivity analyses were performed by additionally adjusting all analyses for log-transformed hsCRP levels, adherence to the gluten-free diet and length of gluten-free diet, and by repeating the analyses with the lifetime major depressive disorder group separated in remitted, partially remitted and current major depressive disorder. Statistical significance was inferred at p <0.05 (two sided). To control for false positive rate due to multiple comparisons a Benjamini-Hochsberg procedure was performed showing that statistical significance could be inferred below the corrected p-value of 0.043. Statistical analyses were performed using SPSS software (Version 19.0. Armonk, NY: IBM Corp).

## Results

### Participant characteristics

No participants had missing data on the main study variables. The patients with CD had a mean age of 55 years (range 20–86 years) and 74% was female (Table 1). Healthy controls had a mean age of 51 years (range 22–66 years) and 67% was female. Both groups had an above average education level. The CD group (n = 77) comprised 44 participants (57%) who had one or more (up to 4) current psychiatric diagnoses. 24 patients (22%) had a current or partially remitted depressive disorder and 14 participants (18%) suffering from any anxiety disorder. CD patients engaged in physical activity 59 MET hours per month less than healthy controls (*p* = 0.01). On average, CD patients were on a gluten-free diet for an uninterrupted period of 15.8 years (SD = 10.4; range 2.5 to 52 years). Length of current gluten-free diet did not differ significantly between never and lifetime major depressive disorder groups. Self-reported diet adherence could be categorized as ‘very strict’ in 69% of participants, ‘strict’ in 30%, and ‘moderately well’ to ‘poor’ in 1%. Diet adherence according to Coeliac Disease Adherence Test results was categorized as ‘excellent’ or ‘very good’ in 63% of participants, ‘very good’ to ‘fair’ in 26% and ‘fair’ to ‘poor’ in 11%.

**Table 1:** Socio-demographic and medical characteristics in celiac disease patients and matched controls.

	Controls	Patients with celiac disease		
	(n = 33)	Never MDD (n = 35)	Lifetime MDD (n = 42)	*P*-value*
Age (years)—mean (SD)	51 ± 13	59 ± 19	52 ± 17	0.13
Gender:—n (%)				
• Male	11 (33.3%)	12 (34.3%)	8 (19.0%)	0.25
• Female	22 (66.7%)	23 (65.7%)	34 (81.0%)	
Level of education:—n (%)				
• Low	7 (21.2%)	8 (22.9%)	10 (23.8%)	0.98
• Intermediate	8 (24.2%)	10 (28.6%)	12 (28.6%)	
• High	18 (54.5%)	17 (48.6%)	20 (47.6%)	
Body mass index (kg/m^2^)—mean (SD)	24.7 ± 3.7	25.1 ± 3.8	24.2 ± 4.0	0.59
Blood pressure:—mean (SD)				
• diastolic (mmHg)	72.7 ± 10.2	72.3 ± 11.7	66.9 ± 8.9^a^ ^b^	0.02
• systolic (mmHg)	121.0 ± 19.8	134.6 ± 20.9^a^	117.5 ± 17.8^b^	<0.001
Current smoker—n (%)	7 (21.2%)	4 (11.4%)	6 (14.3%)	0.52
Alcohol intake:—n (%)				
• no	10 (30.3%)	14 (40.90%)	19 (45.2%)	0.27
• 1–2 u/d	16 (48.5%)	19 (54.3%)	16 (38.1%)	
• ≥2 u/d	7 (21.2%)	2 (5.7%)	7 (16.7%)	
Comorbid medical conditions:—n (%)	1.0 (1.0–2.0)	2.0 (1.0–3.0)^a^	2.0 (2.0–3.0)^a^	0.04
hsCRP (mg/L)	2.62 (0.75–4.48)	1.68 (1.11–2.24)	2.66 (0.33–5.00)	0.70
Physical activity (MET hours/week)	46.2 ± 35.0	31.3 ± 22.3^a^	31.5 ± 27.3^a^	0.048

Data are presented as n (%) or mean (± SD), or median (Q_1_-Q_3_), when appropriate. MDD denotes, major depressive disorder; hsCRP, High-sensitivity C-reactive protein; MET, metabolic equivalents of task.

*: Two sided *p*-values by chi-squared test for categorical variables and by ANOVA for continuous variables.

^a^ Statistically significantly different in post-hoc tests from controls.

^b^ Statistically significantly different in post-hoc tests from never MDD.

### Serum levels of amino acids

All of the tested serum amino acid concentrations differed significantly between CD patients (n = 77) and healthy controls (n = 33), both before and after controlling for the confounders age, gender, education, BMI, alcohol intake, smoking and total energy intake (Table 2). The adjusted mean differences in serum concentrations of LNAA between CD patients and controls were 19.2 μmol/L for valine (95% CI: 7.5–30.8; *p* = 0.002), 6.3 μmol/L for isoleucine (95% CI: 2.3–10.3; *p* = 0.002), and 15.8 μmol/L for leucine (95% CI: 9.1–22.5; *p*<0.001). For the essential amino acids, we found that adjusted mean difference in serum concentrations between CD patients and controls were 6.5 μmol/L for tyrosine (95% CI: 2.0–10.9; *p* = 0.005), 4.1 μmol/L for phenylalanine (95% CI: 1.7–6.6; *p* = 0.001), and 5.8 μmol/L for tryptophan (95% CI: 2.6–9.1; *p* = 0.001). Significant contributions to the models were made by the covariates age, gender, level of education and BMI, with additional contributions by alcohol use and smoking in the analysis of tyrosine.

**Table 2:** Serum amino acid levels in celiac disease patients and matched controls.

	Controls	Patients with celiac disease		
	(n = 33)	Never MDD (n = 35)	Lifetime MDD (n = 42)	*P*-value*
Valine (μmol/L)				
• Crude	221.4 ± 6.0	202.4 ± 4.9^a^	201.1 ± 5.6^a^	0.021
• Adjusted	221.2 ± 4.9	196.7 ± 4.8^a^	206.0 ± 4.3	0.003
Isoleucine (μmol/L)				
• Crude	57.2 ± 2.6	51.3 ± 1.6	49.7 ± 1.7	0.026
• Adjusted	56.9 ± 1.7	49.6 ± 1.7^a^	51.3 ± 1.5^a^	0.007
Leucine (μmol/L)				
• Crude	117.8 ± 3.8	101.9 ± 2.6^a^	100.0 ± 3.1^a^	<0.001
• Adjusted	117.0 ± 2.8	99.6 ± 2.8^a^	102.5 ± 2.5^a^	<0.001
Tyrosine (μmol/L)				
• Crude	60.0 ± 1.7	57.7 ± 2.4	50.4 ± 1.9^a^ ^b^	0.002
• Adjusted	60.0 ± 1.8	56.0 ± 1.8	51.9 ± 1.6^a^	0.006
Phenylalanine (μmol/L)				
• Crude	56.3 ± 0.8	53.0 ± 1.3	52.0 ± 1.0^a^	0.014
• Adjusted	56.5 ± 1.0	51.9 ± 1.0^a^	52.8 ± 0.9^a^	0.004
Tryptophan (μmol/L)				
• Crude	53.9 ± 1.5	47.8 ± 1.6^a^	47.4 ± 1.2^a^	0.003
• Adjusted	53.6 ± 1.4	46.7 ± 1.4^a^	48.6 ± 1.2^a^	0.002

Data are (adjusted) means ± standard error (SE). MDD denotes, major depressive disorder.

*: Two sided *p*-values by AN(CO)VA, adjusted for age, gender, education, BMI, alcohol intake, smoking and total energy intake.

^a^ Statistically significantly different in post-hoc tests from controls.

^b^ Statistically significantly different in post-hoc tests from never MDD.

### Dietary intake of amino acids

There was no significant difference between CD patients and controls in overall energy intake and overall protein intake both before and after controlling for covariates. However, CD patients consumed significantly less vegetable proteins and non-significantly more animal proteins. Vegetable protein intake was significantly lower in CD patients compared to controls (mean 23.7 and 31.5 g/d, respectively; mean difference of 7.8 g/d; 95% CI: 4.7–10.8; *p*<0.001). Although the total amino acid intake and the mean intake of all six tested amino acids was lower in CD patients, none of these differences were statistically significant compared to controls. Correlations between diet intake of individual fatty acids and serum concentrations of fatty acids were low (e.g. Tyr: ρ_s_ = 0.07, *p* = 0.45; Phen: ρ_s_ = 0.11, *p* = 0.24; Trp: ρ_s_ = 0.13, *p* = 0.18).

### Amino acid ratios

The Trp/LNAA ratio based on dietary intake was significantly lower in CD patients than controls, both before and after controlling for covariates (0.046 vs 0.049; mean diff. –0.0025; 95% CI: –0.0033; –0.0016; *p*<0.001). The dietary Trp/BCAA ratio difference was also significantly lower (0.067 vs 0.071; mean diff. –0.0042; 95% CI: –0.0054; –0.0029; *p*<0.001), as was the PhenTyr/LNAA ratio (0.42 vs 0.43; mean diff. –0.010; 95% CI: –0.012; –0.0078; *p*<0.001).

The Trp/LNAA ratio in serum was not different between CD patients and controls, both before and after controlling for covariates (0.10 vs 0.11; mean diff. –0.0011; 95% CI: –0.0085; 0.0063; *p* = 0.77), as was the Trp/BCAA ratio (0.014 vs 0.014; mean diff. –0.0013; 95% CI: –0.012; 0.0090; *p* = 0.81), and the PhenTyr/LNAA ratio (0.26 vs 0.26; mean diff. 0.003; 95% CI: –0.013; 0.019; *p* = 0.72).

### Depression and amino acids

**Fig 3:**
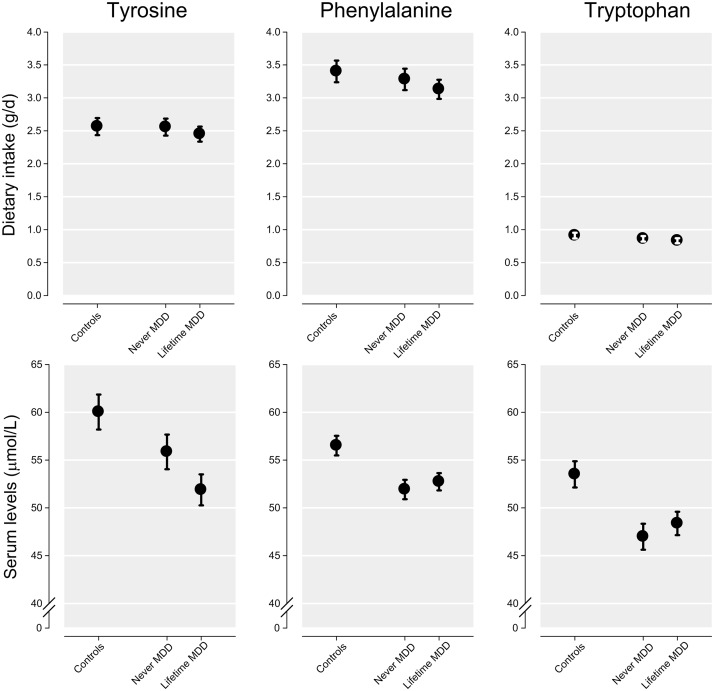
Dietary intake and serum levels of tyrosine, phenylalanine and tryptophan in controls and celiac disease patients with and without depression.

Within CD patients, we also compared the mean amino acid levels and ratios in serum and dietary intake between the never- and lifetime major depressive disorder groups using ANCOVA adjusting for covariates. Overall, amino acid intake and serum values were lower in patients with a lifetime major depressive disorder diagnosis, but they did not differ significantly from values in those without a diagnosis of major depressive disorder (Tables 2 and 3). Results for the three essential amino acids are depicted in Fig 3.

**Table 3:** Diet intake in celiac disease patients and matched controls.

	Controls	Patients with celiac disease		
	(n = 33)	Never MDD (n = 35)	Lifetime MDD (n = 42)	*P*-value*
Total energy intake (kcal/d)				
• Crude	1966 ± 93	2097 ± 112	1864 ± 96	0.25
• Adjusted	1938 ± 103	2091 ± 101	1890 ± 91	0.34
Total protein intake (g/d)				
• Crude	72.6 ± 3.3	69.9 ± 3.4	65.1 ± 3.3	0.27
• Adjusted	71.7 ± 3.4	69.1 ± 3.4	66.4 ± 3.0	0.51
Total vegetable protein intake (g/d)				
• Crude	31.5 ± 1.5	25.7 ± 1.4^a^	22.0 ± 1.0^a^	<0.001
• Adjusted	31.4 ± 1.3	25.4 ± 1.3^a^	22.3 ± 1.1^a^	<0.001
Total animal protein intake (g/d)				
• Crude	41.1 ± 2.4	44.2 ± 2.6	43.2 ± 2.6	0.71
• Adjusted	40.3 ± 2.6	43.8 ± 2.6	44.1 ± 2.3	0.51
Valine (g/d)				
• Crude	4.0 ± 0.2	4.0 ± 0.2	3.7 ± 0.2	0.48
• Adjusted	3.9 ± 0.2	3.9 ± 0.2	3.8 ± 0.2	0.77
Isoleucine (g/d)				
• Crude	3.3 ± 0.2	3.3 ± 0.2	3.1 ± 0.2	0.53
• Adjusted	3.3 ± 0.2	3.27 ± 0.2	3.1 ± 0.2	0.81
Leucine (g/d)				
• Crude	5.8 ± 0.3	5.8 ± 0.3	5.4 ± 0.3	0.47
• Adjusted	5.7 ± 0.3	5.8 ± 0.3	5.5 ± 0.3	0.74
Tyrosine (g/d)				
• Crude	2.6 ± 0.1	2.6 ± 0.1	2.4 ± 0.1	0.47
• Adjusted	2.6 ± 0.1	2.6 ± 0.1	2.4 ± 0.1	0.76
Phenylalanine (g/d)				
• Crude	3.4 ± 0.2	3.3 ± 0.2	3.1 ± 0.2	0.26
• Adjusted	3.4 ± 0.2	3.3 ± 0.2	3.1 ± 0.2	0.46
Tryptophan (g/d)				
• Crude	0.92 ± 0.04	0.87 ± 0.04	0.82 ± 0.04	0.19
• Adjusted	0.91 ± 0.04	0.86 ± 0.04	0.83 ± 0.04	0.40

Data are (adjusted) means ± standard error (SE). MDD denotes, major depressive disorder.

*: Two sided *p*-values by AN(CO)VA, adjusted for age, gender, education, BMI, alcohol intake and smoking.

^a^ Statistically significantly different in post-hoc tests from controls.

### Sensitivity analyses

To take the influence of possible current inflammation into account we performed a sensitivity analysis where we additionally adjusted our multivariate models for log-transformed hsCRP levels. The analysis did not alter the results. We also investigated the relationship between gluten-free diet characteristics and amino acid serum levels and amino acid intake. Both the duration of the gluten-free diet as well as adherence measured by the Celiac Disease Adherence Test did not significantly predict amino acid serum levels, with or without controlling for covariates, nor amino acid intake. Separating the lifetime major depressive disorder group by ‘remitted MDD’, ‘partially remitted MDD’ and ‘current MDD’, yielded essentially similar results.

## Discussion

We investigated the role of amino acid intake and amino acid serum concentrations in depression in patients with CD in remission. CD patients had a lower intake of vegetable protein which may explain the lower serum concentrations of these essential amino acids that we found in CD patients. CD patients also had lower Trp/LNAA ratio and Phen-Tyr/LNAA ratios in their diets compared to controls, pointing to a dietary intake of amino acids that might lead to Trp or Tyr depletion. However, none of the amino acid related findings could be linked to a diagnosis of major depressive disorder in our sample.

Circulating amino acid concentrations are indicative of dietary protein intake and can be used as an indicator of various clinical conditions i.e. malnutrition [38,39]. Intake of dietary protein has been proven to be related to the availability of amino acids in the brain and the effects of amino acid imbalance have been studied extensively, furthermore, imbalanced intake of amino acids such as is used in experimental acute Trp depletion and acute Phen-Tyr depletion studies has been shown to affect mood and cognition [25,40]. Our results show markedly and significantly lower serum concentrations of individual amino acids in CD patients compared to healthy controls and significant lower Trp/LNAA and Trp/BCAA ratios in dietary intake in CD patients compared to controls. In earlier studies lower serum concentrations of monoamine precursors, decreased Trp/LNAA ratio and decreased cerebrospinal fluid levels of serotonin, dopamine, and norepinephrine metabolites have been found in untreated CD patients. These were likely caused by intestinal malabsorption and the situation was assumed to resolve if patients adhere to a lifelong gluten free diet [7,15]. Inflammatory processes however may also lead to reduced Trp availability in patients with CD. A study in 24 children with active CD showed that the serum kynurenine (the first product of tryptophan catabolism) to tryptophan ratio was significantly higher than in controls and as high as in paediatric patients with active Crohn’s disease. Combining this with proof of increased expression of IDO (an anti-inflammatory enzyme that catalyses tryptophan along the kynurenine pathway) in intestinal sections of these CD patients, the authors link intestinal inflammation with increased tryptophan catabolism in active CD [12]. They conclude however that the presence of enhanced IDO expression and enhanced tryptophan metabolism in CD patients in remission remains to be investigated.

In the present study, patients with CD in remission had 11% lower serum Trp concentrations than healthy matched controls. The assumption in earlier studies that improvement of Trp concentrations shortly after the introduction of the gluten free diet points to complete normalisation after a longer period of time [7,11,17] is not supported by our results. Studies following patients for a longer time have up until now not been performed and the question remains if concentrations of monoamine metabolites fully return to normal levels in treated CD. In our study in CD patients on a long term gluten-free diet (mean 16 years) we have found significantly lower serum concentrations of amino acids compared to controls. This finding suggests that brain monoamine availability might be indeed reduced in long term treated CD patients, possibly through low Trp/LNAA or Phen-Tyr ratio or general low Trp or Tyr supply. Acute Trp depletion leads to lower brain availability of serotonin and acute Phen-Tyr depletion leads to lower brain availability of dopamine and norepinephrine [21]. Deficiency of the neurotransmitters serotonin, dopamine and norepinephrine are involved in conditions such as Parkinson’s disease, ADHD, schizophrenia, drug addiction, fibromyalgia and depression [41–43]. In previous studies, a reduction in brain monoamine availability in CD patients was found [7,15]. This reduction may subsequently have induced depression, explaining the increased risk of depression in the CD population as a whole [14]. In contrast to these studies neither intake nor serum levels of amino acids were associated with lifetime major depressive disorder in our CD patients. This might be explained by the small size of our sample and a lack of power to detect an effect.

Several authors have hypothesized that brain function and depression in active CD is linked to intestinal malabsorption through the reduced uptake of amino acids such as tryptophan and consequently a lower production of serotonin [2,7,11,14,16]. Our study gives some support for the idea that in treated CD patients there is malabsorption of these amino acids, since serum concentrations of amino acids were lower in CD patients than in controls while dietary intake of amino acids did not differ. Other mechanism might explain the lower essential amino acid serum concentrations in our sample as well. First, the relative depletion of the essential amino acids (compared to the LNAA, as reflected in their lower ratios) in the diet. Second, poor treatment compliance. However, it is unlikely that this played a major role in our study, since we confirmed longstanding good adherence to the gluten free diet and absence of an active inflammatory process in our patients. Third, the low amino acid serum levels might be related to the prolonged period of the untreated and undiagnosed phase in CD. Destruction of the intestinal lining or microbiome during this phase might have induced a more permanent increase in catabolism and metabolism of (essential) amino acids in CD patients. We suggest this issue needs further study in CD patients [44].

A strength of our study is that it is the first to examine both the amino acid content of the gluten-free diet and serum in CD patients. There are also some limitations that need to be mentioned. Although the differences in our study were statistically significant, they were small and are not likely to produce an immediate clinical effect. Trp loading studies mostly use higher doses, varying from 0.5–7 grams of Trp a day, to produce mood altering effects and improvement in quality of sleep [45,46]. The acute Trp depletion procedure in monoamine depletion experiments however causes a drop in monoamine concentrations of about 70–90% [21]. It is unknown at this point what effect unfavorable amino acid ratio in intake and in serum concentrations will have in the case of a lifelong diet and this low level depletion we found might accumulate its effect over time. We also should consider a possible lack of power in our study, due to the relatively small sample size. Larger studies are necessary to confirm our findings, including prospective studies following patients from the start of the gluten-free diet and over a longer period of time.

In conclusion, we found that CD patients on a long term gluten free diet consume significantly less vegetable protein than controls and have significantly lower serum concentrations of essential amino acid than controls. The clinical significance of the lower amino acid levels is unclear since the findings were not related to depression in our sample.
