# Correction: Essential Amino Acids in the Gluten-Free Diet and Serum in Relation to Depression in Patients with Celiac Disease

**DOI:** 10.1371/journal.pone.0129640

**Published:** 2015-06-03

**Authors:** Nathalie J. M. van Hees, Erik J. Giltay, Susanne M. A. J. Tielemans, Johanna M. Geleijnse, Thomas Puvill, Nadine Janssen, Willem van der Does

In the Author Contributions section, Nathalie J. M. van Hees (NH), Erik J. Giltay (EG), Thomas Puvill (TP), Nadine Janssen (NJ), and Willem van der Does (WD) all should be listed as one of the persons who wrote the paper. The correct contributions are: Conceived and designed the experiments: NH EG WD. Performed the experiments: NH TP NJ. Analyzed the data: NH EG TP NJ WD ST. Contributed reagents/materials/analysis tools: ST JG. Wrote the paper: NH EG WD TP NJ ST JG.
